# Epigenetic context defines the transcriptional activity of canonical and noncanonical NF-κB signaling in pancreatic cancer

**DOI:** 10.1038/s41420-026-03019-9

**Published:** 2026-03-17

**Authors:** Joana E. Aggrey-Fynn, Joshua Busch, Dominik Saul, Ashish Rajput, Kerstin Willecke, Meghana Manjunath, Nicole Klimt, Kothai Rajendran, Nadine Schacherer, Wanwan Ge, Julia Thiel, Amro Abdelrahman, Mark J. Truty, Meng Dong, Steven A. Johnsen

**Affiliations:** 1 Robert Bosch Center for Tumor Diseases (RBCT), Stuttgart, Germany; 2 Department of Biochemistry and Molecular Biology, Mayo Clinic, Rochester, MN USA; 3 Dr. Margarete Fischer-Bosch Institute of Clinical Pharmacology and University of Tübingen, Stuttgart, Germany; 4 Department of Surgery, Mayo Clinic, Rochester, MN USA; 5 University of Tübingen, Tübingen, Germany

**Keywords:** Pancreatic cancer, Transcriptional regulatory elements

## Abstract

NF-κB signaling can be subdivided into canonical and noncanonical pathways, culminating in the transcriptional activity of RELA and RELB, respectively. However, the upstream signals that activate these transcription factors and their specific regulatory roles in pancreatic ductal adenocarcinoma (PDAC) remain incompletely understood. We investigated the differential activation and function of RELA and RELB in PDAC using transcriptome-wide gene expression profiling, genome-wide occupancy mapping, and epigenomic analysis. Temporal activation patterns were assessed following TNFα or TWEAK stimulation. Single-cell RNA sequencing and multiplex immunofluorescence staining were used to characterize activity in primary PDAC tissues. Motif enrichment and chromatin accessibility were evaluated to determine transcription factor binding dynamics and co-regulatory associations. We demonstrate that TNFα is the primary activator of canonical NF-κB signaling via RELA, while TWEAK selectively engages noncanonical signaling through RELB in PDAC. RELA and RELB display distinct temporal dynamics and regulatory activity. RELA binds to both open and closed chromatin and drives a broad transcriptional program, while RELB exclusively occupies pre-accessible chromatin regions co-enriched for AP1 motifs. Motif analysis reveals a particularly strong association of RELB with AP1 elements, suggesting selective co-regulation. Single-cell transcriptomic analysis and multiplex staining in primary tumors reveal distinct spatial and cellular distribution patterns, with RELA and RELB active in separate tumor and microenvironmental compartments. These findings underscore the distinct and complementary roles of TNFα and TWEAK in regulating NF-κB signaling in PDAC. TNFα engages a broader transcriptional program via RELA, whereas TWEAK targets a more selective set of genes marked by chromatin accessibility and AP1 co-binding through RELB. This study provides critical insight into the regulatory dynamics of NF-κB signaling in pancreatic cancer and highlights the specialized functions of RELA and RELB in modulating gene expression and tumor-microenvironment interactions.

## Introduction

The nuclear factor kappa-light-chain-enhancer of activated B cells (NF-κB) signaling pathway is a pivotal regulator of diverse cellular processes, contributing to cancer development and progression. Activation of NF-κB is frequently observed in pancreatic ductal adenocarcinoma (PDAC), where it is associated with aggressive disease. Studies from our lab and others have linked NF-κB activation to oncogenic KRAS signaling and chronic inflammation, driving enhanced survival signaling, tumor growth, metastasis, and therapy resistance [1–5]. Over 70% of PDAC tumors exhibit aberrant NF-κB activity, correlating with poor prognosis, underscoring its role in regulating key aspects of PDAC progression [1, 6, 7].

**Fig. 1:**
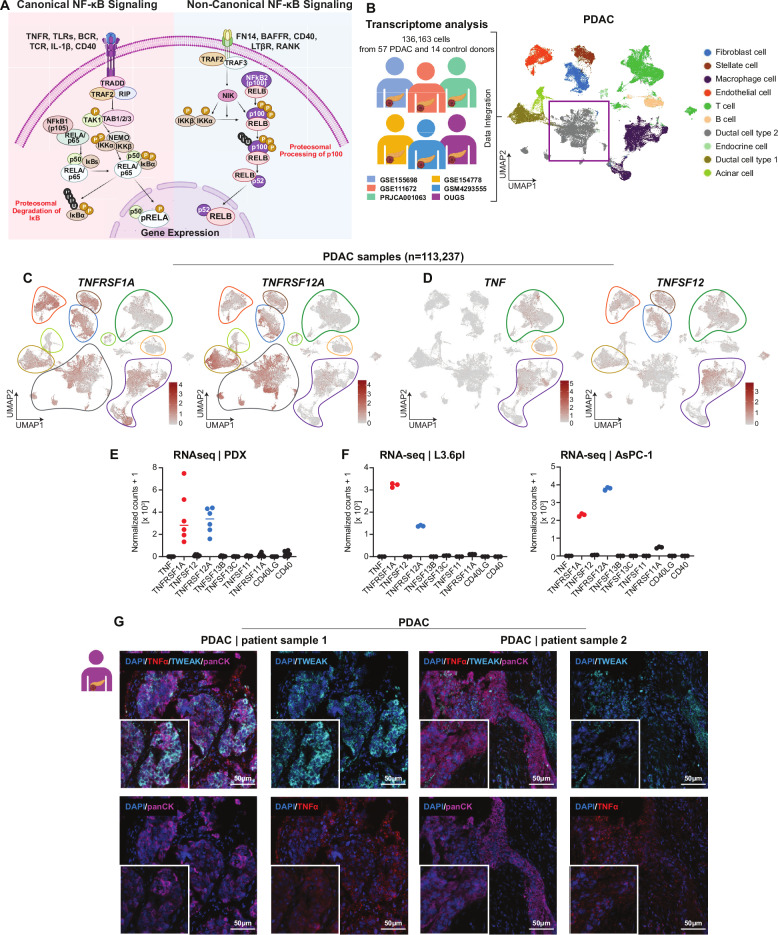
TNFα receptor (*TNFRSF1A*) and TWEAK receptor (*TNFRSF12A*) are the most expressed NF-κB receptors in PDAC. **A** Schematic representation of canonical and noncanonical NF-κB signaling pathways, illustrating their respective ligand-receptor interactions and downstream activation mechanisms. **B** Overview of published scRNA-seq datasets (left) and uniform manifold approximation and projection (UMAP) (*right*) of six scRNA-seq datasets from PDAC patients (*n* = 136,163 cells, *n* = 57 donors, *n* = 14 healthy controls). Cells are colored according to their respective subsets, depicting the cellular landscape of PDAC. **C**, **D** UMAP plots showing the expression patterns of TNFαR (*TNFRSF1A*) and TWEAKR (*TNFRSF12A*) alongside their respective ligands TNFα (*TNF*) and TWEAK (*TNFSF12*), highlighting their distinct distribution across cell populations. **E**, **F** Bar graphs displaying normalized count values for NF-κB pathway receptors and ligands, derived from RNA-seq data from patient-derived xenografts (PDXs) and PDAC cell lines, demonstrating TNFαR and TWEAKR as the most highly expressed NF-κB receptors in these models. **G** Multiplex immunofluorescence staining of PDAC tumor samples showing TNFα (red), TWEAK (cyan), and cytokeratin (pan) (purple), providing spatial localization of NF-κB ligands within the tumor microenvironment. Scale bar represents 50 μm (inserts: 20 μm).

The NF-κB family comprises several members that form distinct dimers and can be divided into two separate signaling pathways referred to as canonical and noncanonical [8–10]. The canonical pathway is activated rapidly by external stimuli, such as the pro-inflammatory cytokine TNFα, through phosphorylation and activation of the IκB kinases (IKKs) [11, 12]. This leads to proteasomal degradation of inhibitory IκB proteins and nuclear translocation of RELA/p50 dimers. The resulting activation is rapid and transient as it simultaneously triggers the expression of negative regulators, including IκBα, A20, and p105, which establish a negative feedback loop [13–15]. In contrast, the noncanonical pathway is slower, relying on the stabilization of NF-κB-inducing kinase (NIK) following degradation of TNFR-associated factor 3 (TRAF3). This allows for the proteasome-dependent proteolytic processing of p100 to p52, releasing RELB/p52 dimers for transcriptional activation [16, 17] (Fig. 1A). While the canonical pathway drives inflammation, immunity, and cell survival, the noncanonical pathway has been implicated in lymphoid development, organogenesis, and sustained immune regulation [9–11, 13, 18].

Recent studies have shown that the traditional binary model of NF-κB signaling, where canonical and noncanonical pathways activate distinct transcriptional effectors, is overly simplistic. Instead, both pathways can regulate overlapping NF-κB dimers, including RELA, RELB, and cREL, depending on the cellular context and stimulus [19]. In this study, we focus on TNFα-induced RELA and TWEAK-induced RELB as representative effectors of the canonical and noncanonical pathways, respectively, to explore how their epigenetic context shapes transcriptional activity in PDAC.

Although the canonical pathway has been extensively studied, less is known about noncanonical NF-κB signaling, and the differential activities of the canonical and noncanonical pathways remain largely unknown. Moreover, the distinct roles of these pathways in PDAC are poorly understood. Based on current literature, canonical and noncanonical pathways likely have complementary but distinct roles in PDAC biology. Thus, understanding these pathways could uncover new therapeutic targets for PDAC treatment.

Using PDAC patient samples and cell lines, we identified tumor necrosis factor-alpha (TNFα) and its receptor (TNFR), and the TNF-like weak inducer of apoptosis (TWEAK) and its receptor (TWEAKR) as key upstream ligand-receptor pairs potentially driving NF-κB signaling in PDAC. Notably, we demonstrate that TNFα-induced RELA is capable of binding both open and closed chromatin regions and inducing H3K27ac at enhancer regions to activate transcription. Conversely, TWEAK-induced RELB binds exclusively to open chromatin regions, relying on other transcription factors such as AP-1 to activate transcription. These findings were validated through multi-omics approaches, including single-cell RNA-seq, mRNA-seq, ChIP-seq, and ATAC-seq, revealing functional and epigenetic distinctions between the canonical and noncanonical pathways.

This work provides novel insights into the regulatory dynamics of NF-κB signaling in PDAC, highlighting pathway-specific differences. These findings could pave the way for approaches to therapeutic interventions targeting canonical and noncanonical NF-κB pathways to improve outcomes in PDAC.

## Results

### TNFα-TNFR and TWEAK-TWEAKR are the most prominent NF-κB signaling pathways active in PDAC

The canonical and noncanonical NF-κB signaling pathways are driven by distinct ligands, receptors, and downstream signaling cascades (Fig. 1A). To identify the major NF-κB signaling pathways active in PDAC, we integrated and analyzed six publicly available single-cell RNA-seq datasets encompassing 57 PDAC and 14 healthy control samples (Fig. 1B). This analysis revealed high expression of *TNF* (TNFα) and its receptor *TNFRSF1A* (TNFR), as well as *TNFSF12A* (TWEAK) and its receptor *TNFRSF12* (TWEAKR) (Fig. 1C, D). Other NF-κB-associated ligands and receptors, such as *TNFSF13B* (BAFF), *CD40*, and *TNFSF11* (RANKL), were also expressed, but at comparatively lower levels (Fig. S1A).

Notably, *TNF* and TWEAK (*TNFSF12*) were more highly expressed in PDAC samples compared to healthy controls (Fig. S1B, C). Furthermore, intercellular communication analysis using CellChat revealed a greater number of inferred interactions in PDAC samples relative to controls (Fig. S1D–F). Signaling pathway-specific analysis suggested increased TWEAK signaling in PDAC tumors versus healthy controls (Fig. S1F). Based on these findings, we sought to further investigate TNF-TNFR and TWEAK-TWEAKR signaling in PDAC.

To further validate these observations, we analyzed RNA-seq data from patient-derived xenografts (PDXs) and PDAC cell lines (L3.6pl and AsPC-1). Normalized counts of the NF-κB pathway ligands and receptors confirmed the predominant expression of the receptors for TNF (*TNFRSF1A)* and TWEAK (*TNFRSF12)* in tumor cells, supporting their potential roles in PDAC (Fig. 1E, F). Multiplex spectral imaging of human PDAC samples corroborated these findings, demonstrating the expression of TNFα and TWEAK in cells within the tumor microenvironment (TME) (Fig. 1G). Together, these results identify TNF-TNFR and TWEAK-TWEAKR as drivers of NF-κB signaling in PDAC.

### Cellular distribution and signaling of TNFα and TWEAK in PDAC

**Fig. 2:**
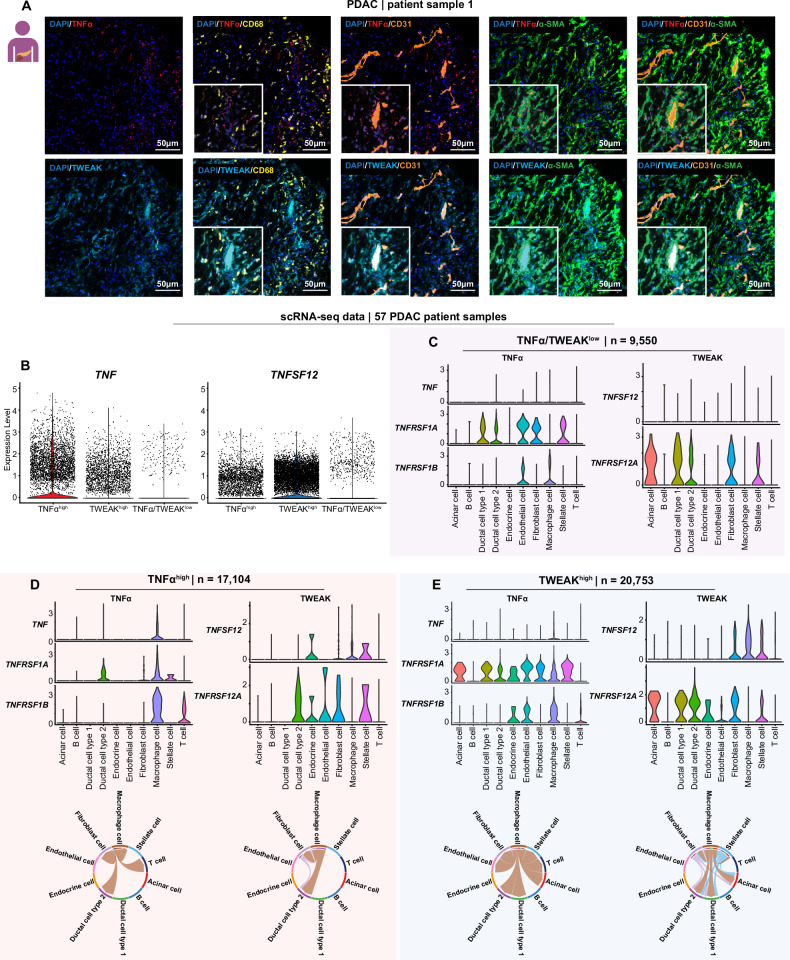
Cellular distribution and signaling networks of TNFα and TWEAK in PDAC. **A** Multiplex immunofluorescence staining of PDAC tumor sample showing TNFα expression enriched in CD68+ macrophage-rich areas (yellow), while TWEAK expression (cyan) overlaps with CD31+ endothelial cells (orange) and α-SMA+ fibroblasts (green), indicating their distinct microenvironmental localization. Scale bar represents 50 μm (inserts: 20 μm). **B** Expression levels of *TNF* (left) and *TNFSF12* right in PDAC patient samples grouped by TNFα/TWEAK^low^ (*n* = 7), TNFα^high^ (*n* = 7 samples), and TWEAK^high^ (*n* = 7) status. Violin plots show the distribution of normalized expression levels. Each dot represents an individual cell. *TNF* expression is highest in the TNFα^high^ group, whereas *TNFSF12* expression is notably higher in the TWEAK^high^ group, reflecting a broader distribution of *TNFSF12*-expressing cells in this population. **C–E** Expression patterns of TNFα and TWEAK ligand-receptor interactions across different cell types, analyzed in TNFα/TWEAK^low^, TNFα^high^, and TWEAK^high^ groups. CellChat analysis was used to compare TNFα and TWEAK signaling strengths, highlighting their differential roles in tumor communication networks.

We next sought to determine the similarities and differences in the cellular source(s) of TWEAK and TNFα in PDAC. Our analysis of the scRNA-seq data revealed that immune cells, mainly macrophages, B cells, and T cells, displayed *TNF* expression. In contrast, while TWEAK was also expressed in a subset of macrophages, it displayed a broader and distinct cellular expression pattern, extending to fibroblasts, endothelial, and stellate cells (Fig. 1D). In general, we observed a higher number of TWEAK expressing-cells compared to TNFα-expressing cells, suggesting it may potentially have a broader effect in PDAC tumors. To further examine and confirm these findings in vivo, we performed multiplex immunofluorescence staining for TNFα, TWEAK, CD31 (endothelial cells), α-SMA (fibroblasts), and CD68 (macrophages). Consistent with our scRNA-seq analyses, we observed TNFα staining primarily in macrophages, while TWEAK was expressed in fibroblasts and endothelial cells, as well as in some macrophages (Figs. 2A and S2A).

To gain biological insight into the effects of TNFα and TWEAK signaling in tumor cells, we stratified PDAC samples into three groups: (1) TNFα/TWEAK^low^ (low expression of both ligands), (2) TNFα^high^ (high TNFα, low TWEAK), and (3) TWEAK^high^ (high TWEAK, low TNFα) (Figs. 2B and S2B–D). CellChat analysis confirmed that each group exhibited distinct ligand-receptor signaling patterns (Fig. 2C–E).

We confirmed that the TNFα/TWEAK^low^ group displayed low expression of both cytokines, and as expected, had minimal network connectivity (Fig. 2C). In the TNFα^high^ group, *TNF*-*TNFRSF1A* and *TNF*-*TNFRSF1B* interactions were predominant, with macrophages serving as the primary source of *TNF* (Fig. 2D). *TNF*-*TNFRSF1A* signaling was directed from macrophages to ductal cell type 2 and fibroblasts, whereas *TNF*-*TNFRSF1B* signaling primarily involved macrophage-T cell interactions (Fig. S2E).

In contrast, the TWEAK^high^ group displayed a broader and more complex signaling network. *TNFSF12* (TWEAK) was highly expressed in macrophages, fibroblasts, and stellate cells. Its receptor, *TNFRSF12A* (Fn14; TWEAKR), was most prominently expressed in ductal cell type 2 and fibroblasts and was present to a lesser extent in macrophages (Fig. 2E). This pattern suggests a primarily paracrine mode of signaling, in which TWEAK produced by macrophages acts on neighboring cells with higher receptor expression. The TWEAK^high^ group exhibited a higher number of inferred interactions and stronger overall signaling activity compared to the TNFα^high^ group, engaging macrophages, fibroblasts, stellate cells, acinar cells, and ductal cells (Fig. S2F).

Collectively, these findings indicate that TWEAK signaling engages a broader range of stromal and epithelial components, likely through paracrine mechanisms, suggesting a more expansive influence of the TME. In contrast, TNFα signaling remains more restricted and immune-centric, with interactions primarily among immune and stromal cells.

### TWEAK induces a limited transcriptional program compared to TNFα

We next sought to characterize the functional signatures associated with TNFα^high^ and TWEAK^high^ cells by analyzing differentially expressed pathways. This revealed an upregulation of pathways involved in translational activity in TNFα^high^ tumor cells and mitochondrial-related processes in tumor cells from TWEAK^high^ tumors (Fig. S3A). Collectively, these findings suggest that tumor cells from TNFα^high^ and TWEAK^high^ tumors display activation of distinct programs in response to signals from the tumor microenvironment.

**Fig. 3:**
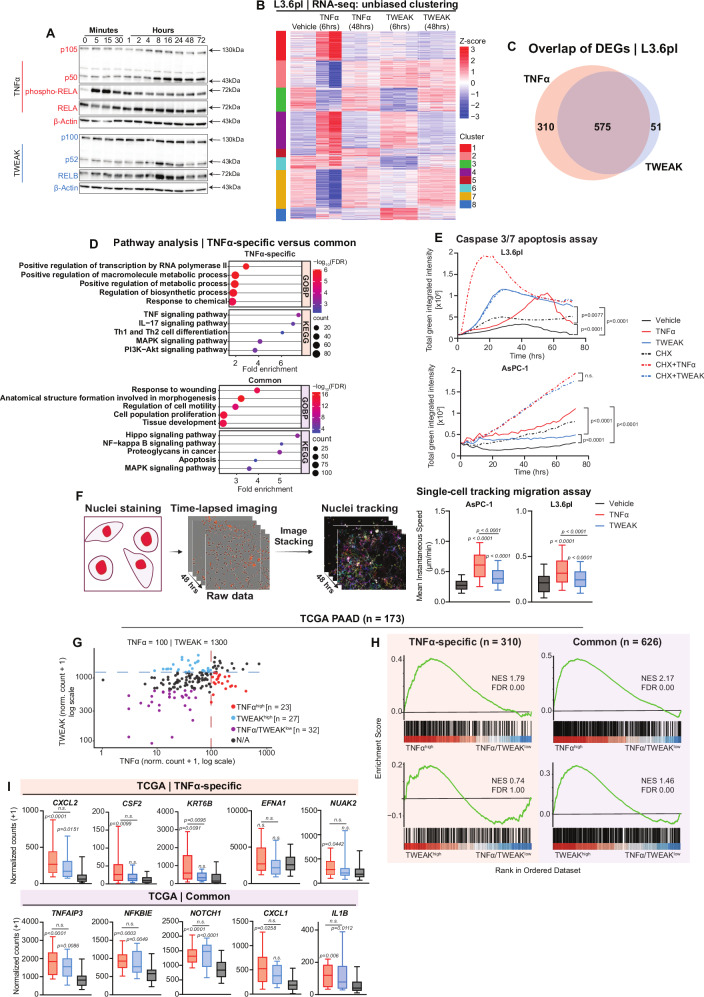
The functional differences and similarities between TNFα and TWEAK signaling activation in PDAC. **A** Western blot analysis of p105/p50, p100/p52, phospho-RELA, and RELB following TNFα and TWEAK treatments across multiple time points (0–30 min and 1–72 h). Representative of *n* = 3 independent experiments. **B** Heatmap of differentially expressed genes following RNA-seq analysis of L3.6pl cells treated with TNFα and TWEAK for 6 and 48 hours. Unbiased clustering analysis was performed after differential expression with DESeq2. One TNFα-treated replicate (replicate 1) was excluded due to low read depth and outlier behavior in PCA and z-score heatmap analyses, which indicated a sequencing error. **C** Venn diagram displaying the number of TNFα-specific, TWEAK-specific, and commonly differentially expressed genes (DEGs), derived from DESeq2 analysis and unbiased clustering analysis. Selections were based on Log2FC ≥ 1, FDR ≤ 0.05, and base mean ≥ 10. **D** Pathway analysis for biological processes (GO) and KEGG of TNFα-specific (top) and common genes (bottom) identified in (**C**). The top pathways were selected based on FDR values. **E** Caspase 3/7 activity in L3.6pl and AsPC-1 cells over 72 h following treatment with TNFα (10 ng/ml), TWEAK (10 ng/ml), and cycloheximide (CHX; 10 µM) as indicated. Values represent total green integrated intensity (normalized to vehicle control and cell number at each time point). Area under the curve (AUC) was calculated for each condition, and *p*-values were determined using unpaired *t*-tests based on AUC values. n.s. = not significant. **F** Single-cell migration tracking assay captured every 15 min over 48 h in AsPC-1 and L3.6pl cells using IncuCyte live-cell imaging (10× magnification). Boxplots depict the mean instantaneous speed calculated from migrated tracks for AsPC-1 and L3.6pl cells following vehicle, TNFα, and TWEAK treatments (*p*-value: one-way ANOVA; Dunnett’s multiple comparisons relative to vehicle control). **G** Scatter plot of normalized expression levels of TNFα and TWEAK (log scale) in 173 PDAC patient samples from TCGA. Each dot represents an individual sample. Based on TNFα and TWEAK expression thresholds (TNFα ≥ 100 and TWEAK < 1300), samples were classified as TNFα^high^ (red), TWEAK^high^ (blue; TNFα < 100 and TWEAK ≥ 1300), TNFα/TWEAK^low^ (purple; TNFα < 60 and TWEAK < 700 or not assigned (black). **H** Gene Set Enrichment Analysis (GSEA) results for TNFα-specific (*n* = 310) and common *(n* = 626; 575 common genes + 51 TWEAK-specific genes) gene sets in the TNFα^high^, TWEAK^high^, and TNFα/TWEAK^low^ expression from TCGA data. Normalized Enrichment Scores (NES) and False Discovery Rate (FDR) values are shown in each plot. TNFα^high^ samples show significant enrichment of both TNFα-specific and common gene sets, whereas TWEAK^high^ samples only display enrichment of the TNFα-specific gene set. **I** Boxplots showing expression of selected genes from TNFα-specific and common gene sets in PDAC samples grouped by TNFα^high^, TWEAK^high^, and TNFα/TWEAK^low^ status. Genes were selected based on their rankings from the GSEA analysis. TNFα-specific genes (top panels) show higher expression in TNFα^high^ samples, while common genes (bottom panels) are elevated in both TNFα^high^ and TWEAK^high^ samples. *p*-values are shown for significant differences (unpaired t-tests).

To further delineate the similarities and differences between canonical and noncanonical NF-κB signaling, we performed transcriptional profiling on the PDAC cell lines AsPC-1 and L3.6pl. First, we assessed the activation dynamics of canonical vs. noncanonical NF-κB signaling by treating PDAC cell lines with TNFα or TWEAK and monitoring RELA, NFκB1 (p105/p50), NFκB2 (p100/p52), and RELB activation. As expected, RELA phosphorylation was detected within 5 min of TNFα treatment, indicating rapid activation of the canonical pathway. In contrast, processing of p105 to p50 was not observed until 8 h post-treatment. This temporal separation suggests that early canonical signaling relies on pre-existing p50, while p105 processing may serve as a delayed, secondary mechanism to sustain or reinforce NF-κB activity in response to TNFα treatment. Like NFκB1, TWEAK-induced p100 processing to p52 and RELB induction also occurred at a later time point (8 h) (Fig. 3A). Together, these findings suggest that while TNFα rapidly activates canonical NF-κB signaling through early RELA phosphorylation, TWEAK-induced p100 processing and RELB induction occur later. This temporal pattern implies that RELA is rapidly induced by TNFα while p52/RELB activation by TWEAK is substantially delayed [10, 20].

To capture early and late response genes, we performed RNA-seq at 6- and 48-h post-treatment in AsPC-1 and L3.6pl cells (Figs. 3B and S3B). Unbiased clustering analysis identified eight gene clusters, where TNFα-specific clusters [1, 5] were enriched in inflammation, immune response, metabolic reprogramming, cell migration, and ECM organization (Fig. S3C, S3D), while shared TNFα/TWEAK clusters [4] were associated with cellular homeostasis, growth, and division (Fig. S3D). Notably, the small TWEAK-dominant cluster [8] exhibited a similar enrichment with TNFα-specific cluster 1 and 5 (Fig. S3D, E), indicating that TWEAK does not drive a distinct transcriptional program in PDAC cells but rather functions to regulate a subset of genes also controlled by TNFα. Additional clusters were linked to cell cycle (cluster 2; Fig. S3F), developmental reprogramming (cluster 3; Fig. S3G), structural organization and metabolism (cluster 6; Fig. S3H), and DNA repair and proliferation (cluster 7; Fig. S3I).

To further confirm the role of TWEAK in inducing a subset of TNFα-regulated genes, we performed differential expression analysis and identified a number of TNFα-specific (*n* = 310) and commonly regulated (*n* = 575) genes, but only a small subset of genes preferentially activated by TWEAK (*n* = 51) (Fig. 3C). These findings demonstrate that TWEAK signaling does not elicit a distinct transcriptional response in PDAC cells but rather modulates core cellular homeostasis and growth pathways also activated by TNFα.

### TNFα and TWEAK both promote apoptosis and migration in PDAC

To further delineate the similarities and differences between TNFα and TWEAK, we analyzed GO enrichment of their shared gene set (Fig. 3C). The overlap indicates that both cytokines regulate genes associated with proliferation, migration, apoptosis, and tissue remodeling. To functionally validate these observations, we examined apoptosis and migration in response to TNFα and TWEAK treatment. Caspase-3/7 activity assays confirmed that the apoptotic machinery was functional in both cell lines following treatment with the positive control staurosporine (Fig. S4A). TNFα and TWEAK both significantly induced low levels of apoptosis in L3.6pl cells, albeit with different time kinetics, but did not appreciably affect apoptosis in AsPC-1 cells (Fig. 3E). As previously described, inhibition of protein synthesis with cycloheximide exacerbates the pro-apoptotic effect of TNF receptor family activation [21]. Consistently, concomitant cycloheximide treatment invoked similar pro-apoptotic effects of TNFα and TWEAK in AsPC-1 cells and facilitated a more rapid and pronounced induction of apoptosis by TNFα in L3.6pl cells, while TWEAK similarly induced apoptosis independent of inhibition of protein synthesis in L3.6pl cells. Collectively, these findings highlight that TNFα and TWEAK are both capable of inducing apoptosis in PDAC cells in a protein synthesis-regulated manner.

In our previous work, we identified an important role for TNFα-induced NF-κB signaling in promoting PDAC cell migration [5]. Thus, we performed single-cell migration assays to compare the effects of TNFα and TWEAK treatment on cell migration. Consistent with our previous work, TNFα significantly enhanced migration in both cell lines, while TWEAK had a significant, but more modest effect (Figs. 3F and S4A).

### TNFα- and TWEAK-associated gene signatures in patient PDAC tissues

To confirm the TNFα-specific and common gene sets we identified from our transcriptional analysis and to assess clinical relevance, we utilized bulk RNA-seq data from TCGA pancreatic cancer samples stratified into TNFα^high^, TWEAK^high^, and TNFα/TWEAK^low^ groups (Fig. 3G). Differential expression analysis revealed that TWEAK^high^ samples exhibited a markedly higher number of upregulated genes (compared to TNFα/TWEAK^low^) than TNFα^high^ samples (1063 vs. 57, respectively), suggesting that TWEAK drives a broader transcriptional response in patient PDAC tumors (Fig. S4B). Consistent with our in vitro studies, we observed significant enrichment of the common gene set in both TNFα^high^ and TWEAK^high^ groups, whereas only TNFα^high^ samples showed significant enrichment of the TNFα-specific gene set (Fig. 3H). Selected genes from these sets further validated these observations, with TNFα-specific genes expressed predominantly in TNFα^high^ samples and common genes enriched in both TNFα^high^ and TWEAK^high^ groups (Fig. 3I). Together, these results validate the TNFα-specific and common gene sets we identified and demonstrate their relevance in human PDAC tissues.

### TNFα and TWEAK differentially activate NF-κB binding dynamics

**Fig. 4:**
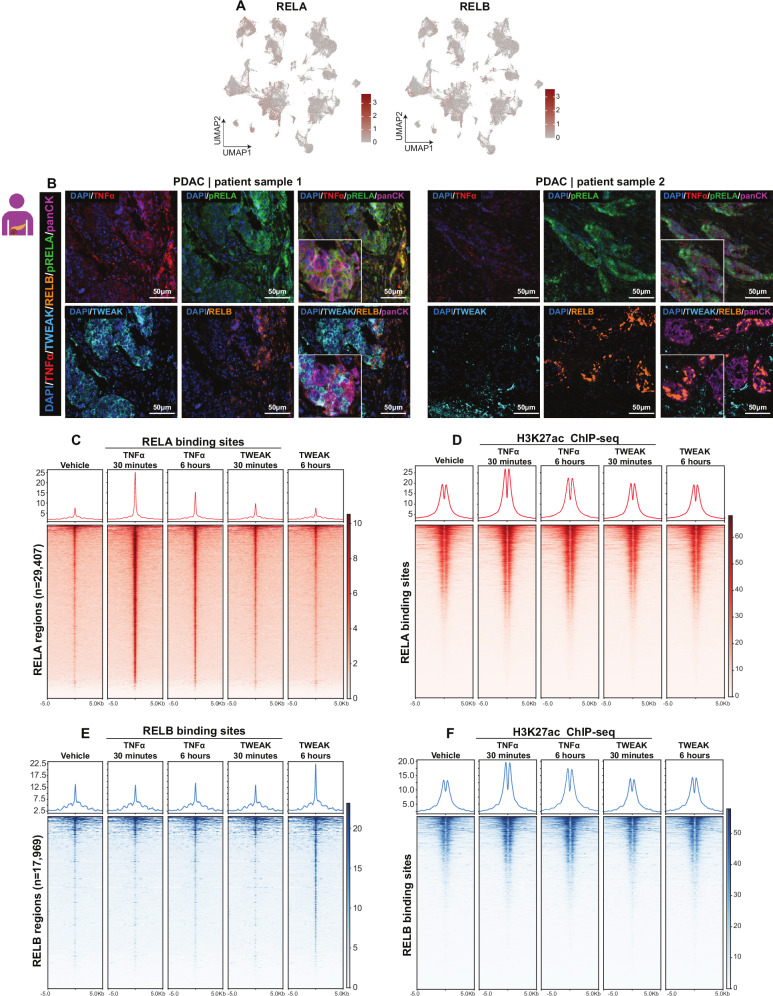
Epigenetic mechanism of canonical and noncanonical NF-κB signaling in PDAC. **A** UMAP plots showing the expression patterns of RELA and RELB in PDAC patient samples (*n* = 136,163 cells, *n* = 57 donors) **B** Multiplex immunofluorescence staining of PDAC patient samples showing phospho-RELA (green) and RELB (orange) expression in TNFα (red) and TWEAK (cyan) expressing cells. Heatmaps and signal intensity profiles of ChIP-seq data for RELA (**C**) and H3K27ac (**D**) across identified RELA binding sites (*n* = 29,407) in PDAC cells. Vehicle-treated and TNFα- or TWEAK-stimulated samples (30 min and 6 h) are shown. RELA ChIP-seq reveals rapid binding of RELA after TNFα treatment (30 min), while TWEAK treatment shows no RELA recruitment. H3K27ac ChIP-seq demonstrates that TNFα treatment induces strong H3K27ac occupancy at RELA sites, with a more robust effect at 30 min compared to 6 h. Heatmaps and signal intensity profiles of ChIP-seq data for RELB (**E**) and H3K27ac (**F**) across identified RELB binding sites (*n* = 17,969) in PDAC cells treated with vehicle, TNFα, or TWEAK for 30 min and 6 h. RELB ChIP-seq shows delayed recruitment in response to TWEAK (6 h) but no significant binding after TNFα stimulation. H3K27ac ChIP- shows minimal changes in H3K27ac occupancy at RELB sites.

Although NF-κB signaling is a well-established mediator of gene activation, the specific roles of the canonical (TNFα) and noncanonical (TWEAK) pathways remain underexplored. To investigate this, we analyzed the genome-wide binding dynamics of RELA and RELB, the respective downstream transcription factors activated by TNFα and TWEAK, respectively (Fig. 1A). scRNA-seq analysis confirmed RELA and RELB expression in PDAC cells (Fig. 4A). Notably, multiplex immunofluorescence staining in PDAC patient samples confirmed that activation of RELA (via phosphorylation) and expression of RELB in epithelial tumor cells (expressing cytokeratin) are closely associated with the expression of the respective ligand in the proximal TME in patient tumor samples (Fig. 4B).

To investigate the temporal recruitment of RELA and RELB to chromatin and associated epigenetic changes, we performed ChIP-seq for RELA, RELB, and H3K27ac following TNFα or TWEAK treatment for 30 min and 6 h, based on activation kinetics observed in Fig. 3A. TNFα treatment induced selective RELA (but not RELB) binding at early time points, accompanied by an increase in adjacent H3K27ac occupancy (Fig. 4C, D), while TWEAK treatment selectively induced RELB (but not RELA) binding at a later time point without notably increasing H3K27ac at these sites (Fig. 4E, F). These findings demonstrate that TNFα specifically activates RELA and promotes H3K27ac enrichment at NF-κB-associated regions, whereas TWEAK selectively activates RELB without significantly altering H3K27ac occupancy.

### TNFα induces broader NF-κB binding and epigenetic activation than TWEAK

**Fig. 5:**
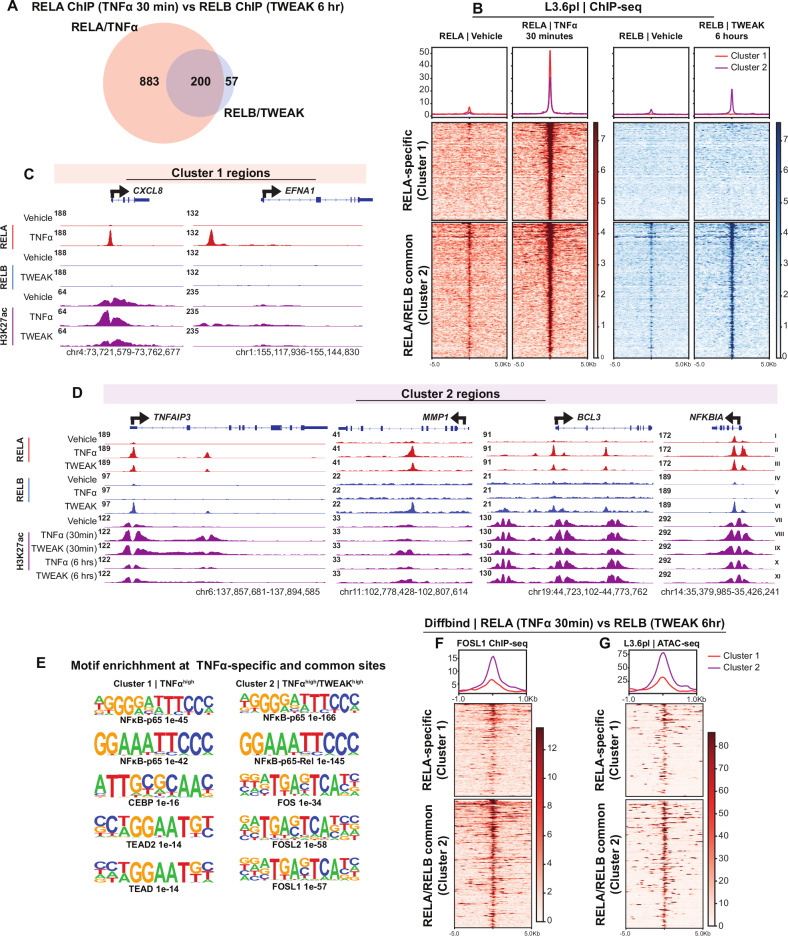
RELA/RELB common regions are associated with chromatin accessibility and AP-1 factors. **A** Venn diagram comparing RELAc and RELB bound regions identified by DiffBind and bedtools intersect analysis. RELA binding was assessed 30 min after TNFα treatment, while RELB binding was assessed 6 h after TWEAK treatment. The regions were selected based on: RELA/TNFα-specific regions (Log2FC ≥ 1, FDR ≤ 0.05). RELB/TWEAK-specific regions (FDR ≤ 0.05, with only 57 regions differentially bound). Common regions. **B** Heatmap of RELA at the two defined clusters. Cluster 1 includes the top 150 regions (from 883 RELA-bound TNFα-induced regions) selected based on the highest binding intensity using DiffBind. RELA and RELB signals for the 57 TWEAK-specific regions were included in the common regions (Cluster 2; *n* = 257) following vehicle, TNFα, and TWEAK treatments, reflecting shared regulatory elements between canonical and noncanonical NF-κB signaling. Genome browser (IGV) tracks of ChIP-seq data in L3.6pl cells showing RELA, RELB, and H3K27ac signals at: RELA/TNFα-specific gene loci (**C**; *CXCL8* and *EFNA1*) and Common gene loci (**D**; *TNFAIP3*, *MMP1*, *BCL3*, and *NFKBIA*). Tracks illustrate RELA, RELB, H3K27ac signals following vehicle, TNFα, and TWEAK treatments, highlighting distinct and overlapping regulatory regions of RELA and RELB. **E** Top enriched motifs identified in Cluster 1 (RELA-specific regions) and Cluster 2 (RELA/RELB common regions) using HOMER motif analysis, demonstrating distinct transcription factor binding preferences. **F** Heatmap of FOSL1 signals across Cluster 1 (RELA-specific) and Cluster 2 (RELA/RELB common regions), showing its preferential binding patterns. **G** ATAC-seq signal intensity at Cluster 1 and Cluster 2 in L3.6pl cells, indicating differences in chromatin accessibility between RELA-specific and RELA/RELB common regions.

To investigate transcriptional regulatory mechanisms controlled by canonical and noncanonical NF-κB signaling, we performed differential binding analysis (DiffBind) comparing RELA and RELB binding at 30 min following TNFα treatment and 6 h after TWEAK treatment, respectively. This analysis revealed that RELA bound significantly more regions than RELB (Figs. 5A and S5A). Consistent with our RNA-seq results where TNFα induced a distinct subset of genes, while TWEAK-induced a subset of genes that was also induced by TNFα (Fig. 3C), RELB-bound regions were also bound by RELA, while many RELA-bound regions displayed no significant enrichment of RELB (Fig. 5A, B). Thus, we could separate RELA/RELB-bound regions into cluster 1, containing RELA-specific regions, and cluster 2, containing commonly bound regions. Cluster 1 included the top 150 TNFα-specific regions, selected from the larger set of 883 TNFα-unique peaks based on binding intensity using DiffBind, and was used to represent RELA-specific binding. Cluster 2 comprised the 200 regions shared between TNFα and TWEAK, combined with the 57 TWEAK-specific regions (totaling 257), representing commonly bound and TWEAK-enriched sites (Fig. 5B–D). This clustering strategy allowed us to distinguish regions preferentially bound by RELA in response to TNFα from those co-occupied or more responsive to TWEAK and RELB.

Analysis of H3K27ac occupancy at these regions confirmed the more pronounced effect of TNFα-activated RELA in promoting H3K27 acetylation at NF-κB-associated loci (Fig. S5B). To extend our analysis beyond H3K27ac, we examined H3K4me1, H3K4me2, and H3K4me3 enrichment under basal conditions and observed a similar pattern, with consistently higher signal at regions shared between TNFα and TWEAK responses (Fig. S5C). These findings support the idea that the common regions exist in a primed chromatin state prior to stimulation. Together, these findings demonstrate that TWEAK-induced noncanonical NF-κB selectively promotes RELB binding at epigenetically primed genomic regions that make up a subset of regions bound by RELA after activation of canonical NF-κB signaling by TNFα treatment. Additionally, TNFα stimulated RELA binding to a number of additional regions selectively bound by RELA, but not RELB, which also display enhanced H3K27ac occupancy following ligand treatment. This differential regulation underscores the distinct yet overlapping contributions of TNFα and TWEAK in shaping NF-κB-dependent epigenetic activation of transcription.

### RELB relies on chromatin accessibility and AP-1 factors for gene regulation

To further explore the regulatory mechanisms associated with the differential transcriptional effects elicited by RELA and RELB downstream of TNFα and TWEAK signaling, respectively, we examined transcription factor enrichment on the RELA-specific genomic regions (cluster 1) as well as the RELA/RELB-common regions (cluster 2) (Fig. 5B). Cluster 1 was primarily enriched for RELA and NFκB1, while in addition to enrichment for REL factors, cluster 2 also displayed a significant enrichment of AP-1 factors (e.g., FOS and JUN families of AP-1) (Fig. S5C). Similarly, motif enrichment analysis confirmed the presence of RELA (NFκB-p65) and TEAD motifs in cluster 1, whereas cluster 2 displayed a significant enrichment of AP-1 motifs in addition to RELA motifs (Fig. 5D). These results suggest that TNFα-induced RELA is able to largely function independently, while TWEAK-induced RELB may require additional transcription factors, such as AP-1 factors, for activity.

To further test this, we examined FOSL1 occupancy on cluster 1 (RELA-specific) and cluster 2 (RELA/RELB common regions). Intersection analysis revealed that 77% of RELB regions overlapped with FOSL1 (Fig. S5D) while just 27% of RELA-occupied regions intersected with FOSL1. Our previously published data [5] indicated that RELA-FOSL1 co-bound regions regulate cell migration in PDAC through the convergence of MAPK and NF-κB signaling pathways. Consistently, FOSL1 binding was higher in common regions (Cluster 2) than in RELA-specific regions (Cluster 1) (Figs. 5F and S5E).

We next sought to test whether the differences in RELA and RELB binding may be related to differences in chromatin accessibility prior to pathway activation. Thus, we examined chromatin accessibility by ATAC-seq [22] and observed that common regions (Cluster 2) exhibited significantly higher chromatin accessibility compared to RELA-specific regions (Cluster 1) (Figs. 5G and S5E). These findings underscore that noncanonical TWEAK signaling through RELB relies heavily on an open chromatin state to regulate gene expression, while TNFα-driven RELA binding can occur even at closed chromatin regions. Together, our results reveal that TNFα-driven RELA autonomously binds and regulates gene expression by increasing H3K27ac and activating enhancer regions, while TWEAK-induced RELB relies on the binding of other factors, such as AP-1, to provide an open chromatin conformation and active epigenetic state. Thus, these two pathways function with partially overlapping, but distinct mechanisms that may be useful for dissecting their individual contributions to cancer biology and potentially targeting individual aspects of NF-κB biology.

## Discussion

NF-κB signaling is a key driver of PDAC, with persistent activation observed in 70% of tumors [1, 23]. While the canonical (TNFα-TNFR) NF-κB pathway has been extensively studied, significantly less is known about the noncanonical (TWEAK-TWEAKR) pathway or the functional and differential epigenetic mechanisms by which these two pathways function in PDAC. Through multi-omics approaches, we uncover divergent chromatin-binding dynamics of RELA and RELB, highlighting the ability of TNFα to remodel chromatin and autonomously activate NF-κB-dependent transcription through RELA, while TWEAK-driven RELB activation requires pre-existing chromatin accessibility and co-factors such as AP-1.

Our results demonstrate that TNFα rapidly activates canonical NF-κB signaling, with RELA driving the expression of a vast number of genes. Functionally, TNFα-induced apoptosis is significantly enhanced when translation is inhibited, suggesting a dependency on rapidly synthesized survival factors such as MCL-1 and XIAP [24–26]. In contrast to the rapid transcriptional effects of TNFα, TWEAK-induced noncanonical NF-κB signaling exhibits slower activation dynamics and lacks a unique transcriptional program in PDAC cells, with nearly all induced genes also being induced by TNFα.

Our scRNA-seq analysis revealed that TWEAK is expressed by a broader range of cell types in PDAC, whereas TNFα is predominantly expressed by immune cells. Consistent with this, CellChat-based interaction analysis showed that TWEAK has greater cumulative signaling strength and a more complex network of ligand-receptor interactions. Consistent with a broader expression of TWEAK in the TME, TCGA analysis identified more drastic transcriptional differences in TWEAK^high^ tumors compared to TNFα^high^ tumors, suggesting that the TWEAK signal produced by more cells in the TME is able to engage more individual tumor cells and elicit an overall stronger transcriptional response in bulk RNA-sequencing. This finding is consistent with our multiplex immunofluorescence staining showing a broad expression of TWEAK in the PDAC TME. Interestingly, in contrast to the in vivo data, TNFα stimulation of PDAC cell lines in vitro induced a more robust transcriptional response than TWEAK. Thus, these findings support a model in which TWEAK recapitulates many of the transcriptional effects of TNFα, but through a slower, broader, and more distributed signaling program within the TME. This is likely driven by TWEAK’s expression in diverse stromal and epithelial compartments. In contrast, TNFα signaling elicits a significantly stronger effect but is more tightly controlled through interactions with specific components of the TME.

NF-κB is known to shape the epigenetic landscape in cancer by influencing chromatin accessibility, histone modifications, and transcription factor recruitment [1, 4, 27]. Our ChIP- and ATAC-seq analyses reveal key differences in RELA and RELB chromatin binding and activation dynamics. TNFα-induced RELA is able to bind to both open and closed chromatin regions and to establish active enhancers by promoting H3K27ac. This suggests that TNFα-driven RELA activation is sufficient to remodel chromatin and initiate transcription, reinforcing its autonomous role in NF-κB-driven oncogenic programs. In contrast, TWEAK-induced RELB binding is largely restricted to regions that are already accessible and marked by H3K27ac, reflecting its reliance on a primed chromatin state and collaborating factors such as AP-1. Unlike RELA, RELB does not autonomously remodel chromatin, underscoring distinct regulatory capacities of canonical and noncanonical NF-κB pathways. Future work evaluating TNFα-dependent translational processes and TWEAK-dependent effects on mitochondrial activity, ROS production, and related metabolic effects will be essential for deeper mechanistic dissection of how these distinct regulatory pathways shape downstream cellular responses.

The identification of TNFα and TWEAK as dominant NF-κB signaling axes in PDAC presents potential therapeutic opportunities. Given the ability of TNFα to activate genes controlled by both canonical and noncanonical NF-κB pathways, strategies specifically targeting transcriptional mechanisms controlling these genes (e.g., chromatin remodeling) could potentially disrupt PDAC progression. Furthermore, the reliance of TWEAK-induced RELB on chromatin accessibility, AP-1, and possibly other cofactors suggests potential vulnerabilities that could be exploited using inhibitors of chromatin remodeling or transcription factor modulators. AP-1 has been implicated in pancreatic cancer invasion and metabolic reprogramming, making it a potential target for combination therapies with NF-κB inhibitors [28, 29]. Targeting the NF-κB/AP-1 axis, either through chromatin remodeling inhibitors or MAPK blockade, could disrupt tumor adaptation mechanisms. Together, these findings show that TNFα and TWEAK engage NF-κB through distinct chromatin-based mechanisms, offering complementary avenues for therapeutic intervention in PDAC.

Our study provides a comprehensive analysis of TNFα- and TWEAK-mediated NF-κB signaling in PDAC, revealing distinct transcriptional, epigenetic, and functional differences. Our data suggest that TNFα is a primary driver of inflammation, metabolic adaptation, and migration through direct chromatin remodeling and autonomous RELA activity, whereas TWEAK relies on chromatin accessibility and transcriptional co-factors such as AP-1 to regulate gene expression. These findings suggest that TNFα-driven functions in PDAC may be differentially vulnerable compared to TWEAK-driven effects, which may be targeted by disrupting NF-κB/AP-1 interactions. Moreover, these results provide important new insights into the similarities and differences between canonical and noncanonical NF-κB and reveal important differences in the effects elicited by different components of the TME on PDAC tumor cell gene expression. A comprehensive evaluation of additional canonical and noncanonical ligands will be an important future direction to determine the extent to which these mechanisms generalize across the broader NF-κB signaling landscape. Further studies will be necessary to elucidate how these mechanisms can be exploited for improved patient therapy.

### Compliance with ethics statement

This study received all necessary ethical approvals from the Institutional Review Board (IRB) at the Mayo Clinic under protocols [354-06, 66-06, and 19-012104]. All experimental procedures and methods were conducted in accordance with relevant institutional guidelines and regulations. Informed consent was obtained from all human participants or their legal guardians prior to inclusion in the study, in accordance with IRB-approved protocols and institutional standards.

## Materials and methods

### Cell culture

AsPC-1 (RRID:CVCL_0152) cells were maintained in RPMI 1640 Medium (Corning). L3.6pl (RRID:CVCL_0384) cells were maintained in phenol red-free minimum essential media (MEM; Thermo Fischer Scientific). Media were supplemented with 10% FBS (Corning), 1% Penicillin/streptomycin (Thermo Fischer Scientific), and 1% L-Glutamine (Corning, for MEM media). Cells were split upon reaching 70–80% confluence. All treatments were performed in the appropriate media, and the list of proteins and inhibitors and concentrations used is provided in Supplementary Table S1. Cells were treated with TNF (10 ng/ml; R&D systems 210-TA-100), TWEAK (10 ng/ml; R&D systems 1090-TW-025), caspase 3/7 (1:500; ThermoFisher; C10432), and cycloheximide (10 µM; Sigma Aldrich; C7698).

### Protein isolation, RNA-seq, and ChIP-seq library preparation

Protein isolation and western blots were performed as previously reported [30]. The following primary antibodies were used at the indicated dilutions: phospho-NFκB p65 (S536) (93H1) (phospho-RELA; 1:1000; Cell Signaling Technology; 3033; RRID:AB_331284), RELB (D7D7W) (1:1000; Cell Signaling Technology; 10544; RRID:AB_2797727), NF-κB2 p100/p52 (18D10) (1:1000; Cell Signaling Technology; 3017; RRID:AB_10697356), NF-κB1 p105/p50 (D4P4D) (1:1000; Cell Signaling Technology; 13586; RRID:AB_2665516), and β-Actin (D6A8) (1:1000; Cell Signaling Technology; 8457; RRID:AB_10950489). Secondary antibodies were used at a 1:2500 dilution and included goat anti-rabbit IgG Starbright Blue 700 (Bio-Rad; 1200416; RRID:AB_2721073) and goat anti-mouse IgG Starbright Blue 520 (Bio-Rad; 12005866; RRID:AB_2934034).

For the RNA-seq library, RNA quality was validated by gel electrophoresis. We used 500 ng to make the libraries in triplicate for each condition. Libraries for cells were made using the TruSeq RNA Library Prep Kit V3 (Illumina) according to the manufacturer’s instructions. ChIPs and ChIP-seq were performed as previously described with minor changes [31, 32]. Libraries were prepared using the MicroPlex Library Preparation Kit v2 (Diagenode) according to the manufacturer’s protocol. Details protocol for RNA- and ChIP-seq are provided in Supplementary information. DNA quality of the resulting DNA was measured using the High Sensitivity DNA Kit (Agilent) on the Agilent TapeStation 4150 (RRID:SCR_019393). Antibodies used for ChIP are provided in the ChIP-seq methods section below. Samples were sequenced (paired-end 50 bp) on a NextSeq 2000 sequencer (P2, Illumina; RRID:SCR_023614) at the Robert Bosch Center for Tumor Diseases (RBCT).

### Cell migration analysis from IncuCyte time-lapse imaging

A total of 3000 cells were seeded and treated overnight with Nuclight Red dye (1:2000; Sartorius). The cells were then treated with proteins and inhibitors as specified in the cell culture section above. Live cell imaging was performed using the Sartorius IncuCyte (RRID:SCR_023147). Images were captured every 15 min for 48 h and processed using the Basic Analyzer tool (Sartorius). Preprocessing and image analysis were performed blots were performed as previously reported [5].

Box plots were plotted using the 10th to 90th percentile range. The error bars represent the 10th and 90th percentiles of the data, reflecting the spread of the central 80% of values. These are percentile-based and do not correspond to standard deviation (s.d.), standard error of the mean (s.e.m.), or confidence intervals (c.i.).

### scRNAseq data analysis for publicly available datasets

For the single cell RNAseq analysis, the following publicly available PDAC patient datasets were downloaded from the GEO database (RRID:SCR_005012), GSE154778 [33], GSE111672 [34], PRJCA001063 [35], GSE155698 [36], GSM4293555 from GSE141017 [37], and scRNA-seq data from the study by Chijimatsu et al., 2022 [38]. The processed data for PRJCA001063 [35] were obtained from zenodo [10.5281/zenodo.3969339] (RRID:SCR_004129), which included cell label annotations for 10 cell types after quality control (QC) steps. Other scRNA-seq datasets were downloaded from the NCBI GEO database or as specified in their respective publications. These datasets were combined, harmonized (harmony v0.1), and prepared for downstream analysis using the bioinformatics methodology described by PMID: 31740819 [39].

Seurat objects (RRID:SCR_022555) were created for individual datasets by reading the Cellranger output files into the R environment using the Read10x function and transforming them using the CreateSeuratObject function, as described in the Seurat Guided Clustering Tutorial (https://satijalab.org/seurat/articles/pbmc3k_tutorial). The analysis was conducted using R version 4.4.2 and Seurat version 5.2.01. Transcript counts, measured as UMIs, were normalized to 10,000 counts per cell and log-transformed, following the methodology described by Chijimatsu et al. [38]. Cells with a high percentage of mitochondrial genes (>25%) were filtered out during the QC steps. Other QC metrics for individual datasets, such as UMI counts and the number of expressed genes, were also applied as per Chijimatsu et al. [38].

Datasets were batch-corrected and integrated using the rPCA method as outlined in the Seurat package (https://satijalab.org/seurat/articles/integration_rpca.html). Each dataset was scaled, and the FindVariableFeatures function was employed to identify highly variable genes. These genes were utilized for PCA analysis (RRID:SCR_014676). An anchor was created using the FindIntegrationAnchors function with the following parameters: 30 principal components, rPCA, and two reference datasets (PRJCA001063 and GSE155698). Subsequently, six datasets were integrated using the IntegrateData function of the Seurat package. The integrated dataset was scaled, followed by PCA analysis and UMAP (RRID:SCR_018217) visualization. Cell-type annotation was transferred from the reference dataset PRJCA001063 [35]. CellChat analyses were conducted using CellChat (v1.5.0, PMID: 39289562). The heatmaps were designed after manual selection of the topgenes and normalizing as scaling using the DoHeatmap function in Seurat (v5.2.0). The gene enrichment was performed using bitr and enrichGO from the clusterProfiler (4.14.4) and enrichplot (1.26.6) packages and plotted with the ggplot2 package (3.5.2).

### mRNA-seq

The integrity of RNA was validated by gel electrophoresis, and 500 ng was used to make the libraries in triplicate for each condition. Libraries for cells were made using the TruSeq RNA Library Prep Kit V3 (Illumina) according to the manufacturer’s instructions. Oligo-dT beads were used to capture poly-A tailed-mRNA followed by first-strand cDNA synthesis by Superscript II reverse transcriptase (Thermo Fischer). Second-strand synthesis was followed by end repair, 3′ adenylation, adaptor ligation, and library amplification. Agencourt AMPure XP (Beckman Coulter) was used for size selection during the library synthesis. The quality of the resulting DNA was measured with high sensitivity DNA kit (Agilent) on the Agilent TapeStation 4150. Samples were sequenced (paired-end 50 bp) on a HiSeq4000 (Illumina) at the Genome Analysis Core at the Mayo Clinic (30-min-treated RNA-seq samples) and on a NextSeq 2000 (P2, Illumina) at the Robert Bosch Center for Tumor diseases (RBCT).

### RNA-seq bioinformatic analysis

Bam files were generated using STAR version 2.7.3a (RRID:SCR_004463) [40]. Features were counted using HTSeq version 0.9.1 (RRID:SCR_005514) [41]. Differential gene expression analysis was performed by DESeq2 (RRID:SCR_015687) [42]. Upregulated genes were identified as ≥1 log2 Fold Change, FDR≤0.05, and BaseMean≥10. Pathway analyses were performed using ShinyGO version 0.80 (RRID:SCR_019213) [43].

### RNA-seq quality control and sample exclusion

For both AsPC-1 (Fig. S3b) and L3.6pl (Fig. 3b) cell lines, one RNA-seq sample from the TNFα-treated group (replicate 1) was excluded from downstream analysis. This decision was based on pre-analysis quality control, which included evaluation of read counts, principal component analysis (PCA), and z-score heatmaps. In both cases, the excluded sample exhibited markedly low total read counts and clustered as an outlier in PCA plots. Further investigation revealed the issue was due to sequencing error. Although the exclusion criteria were not pre-established, they were applied consistently based on these objective quality metrics.

### TCGA Data Acquisition

For RNA-seq data analysis, Gene expression data for pancreatic adenocarcinoma (PAAD) were obtained from the TCGA-PAAD project via the UCSC Xena Browser (https://xenabrowser.net, RRID:SCR_005012). The dataset TCGA-PAAD.star_counts.tsv, containing STAR-aligned gene-level expression in log2(count + 1) format, was downloaded from the GDC Xena Hub (https://gdc.xenahubs.net). In addition, clinical phenotype metadata were also obtained from the GDC data portal (RRID:SCR_014514) for sample annotation and filtering. The TCGA-PAAD dataset includes both primary tumor and normal pancreatic tissue samples and is publicly available under the accession number phs000178 in the dbGaP database (https://www.ncbi.nlm.nih.gov/projects/gap/cgi-bin/study.cgi?study_id=phs000178).

Sample selection was performed following rigorous preprocessing, including removal of normal tissue samples (barcode suffix −11A), tumor subtypes such as cystic, mucinous, and serous adenocarcinomas, and samples with low or missing expression of TNF and TWEAK. Gene identifiers were cleaned by removing Ensembl version suffixes and mapped to HGNC gene symbols using the biomaRt package (RRID:SCR_002987). After quality control, 82 primary tumor samples and 23,495 genes were retained for downstream analysis. Samples were then categorized into three expression groups; TNF^high^, TWEAK^high^, and TNF/TWEAK^low^, based on data-driven expression thresholds for TNF and TWEAK genes, determined using summary statistics of their expression distributions.

### TCGA bioinformatic analysis

Differential gene expression was assessed using the limma package (RRID:SCR_010943) [44] from Bioconductor. The input matrix, already log2(count + 1) transformed, was quantile normalized using normalizeBetweenArrays() to reduce technical variability. Limma was selected over count-based methods such as DESeq2 or edgeR due to the log-transformed nature of the input data and its robust performance with small-to-moderate sample sizes. Group comparisons were defined based on TNF and TWEAK expression levels. A design matrix without intercept ( ~ 0 + group) was used to fit group-specific linear models with lmFit(). Empirical Bayes moderation was applied using eBayes(trend = TRUE) to account for the mean-variance relationship typical of RNA-seq data.

Genes were considered significantly differentially expressed if they met the criteria: adjusted *p*-value < 0.05, |log2 fold change| > 1, and average expression ≥ 5 (log2 scale). Significant genes were saved separately for downstream visualization and enrichment analysis.

### Gene set enrichment analysis (GSEA)

GSEA was conducted using the clusterProfiler package (RRID:SCR_016884) [45]. Gene lists ranked by log2 fold change were analyzed using the GSEA() function, which internally uses the fgsea algorithm (RRID:SCR_020938) [46] for fast enrichment scoring. Custom gene sets, such as TNFα_specific and Common_Genes (supplementary materials), were prepared in GMT format and loaded with read.gmt(), then passed via the TERM2GENE argument. The analysis used the following parameters; pvalueCutoff = 1, minGSSize = 1, maxGSSize = 600. Documentation for the GSEA function is available in the clusterProfiler user manual [46]. Enrichment statistics, including normalized enrichment score (NES), nominal *p*-values, and leading-edge genes, were computed. Selected gene sets were visualized using the plotEnrichment() function, and results were saved in TSV and PDF formats for reporting.

Analyses were conducted in R v4.3.1 using the following packages: limma v3.60.6, clusterProfiler v4.12.6, fgsea v1.30.0, and biomaRt v2.60.1.

### Chromatin immunoprecipitation sequencing (ChIP-seq)

Chromatin immunoprecipitation was performed as previously described [32, 47]. Antibodies included H3K27ac (1μg; C15410196, Diagenode; RRID:AB_2637079), FOSL1 (D80B4) (FRA-1) (5 μl; 5281; Cell Signaling; RRID:AB_10557418), RELA (NFκB p65 L8F6) (5 μl; 6956; Cell Signaling; RRID:AB_10828935), RELB (5 μl; Cell Signaling; 10544; RRID:AB_2797727), and control Rabbit IgG (1 μg; C15410206, Diagenode; RRID:AB_2722554). Protein A-Sepharose and protein G-Sepharose (for RELA) beads were added to the samples and incubated for 2 h, washed, de-crosslinked, and DNA was extracted. Samples were performed in triplicate for each. Condition. Libraries were prepared using the MicroPlex Library Preparation Kit v2 (Diagenode) according to the manufacturer’s protocol. DNA integrity was measured with a high-sensitivity DNA kit (Agilent) on the Agilent TapeStation 4150. Samples were sequenced (paired-end 50 bp) on a NextSeq 2000 (P3, Illumina) at the Robert Bosch Center for Tumor Diseases.

### ChIP-seq bioinformatic analysis

Reads were mapped to the reference genome assembly (hg38) by BOWTIE2/2.5.0 (RRID:SCR_016368) [48]. Bigwig files were generated from merged bam files using bamCoverage (RRID:SCR_016366). Localization profiles were viewed using Integrative Genomics Viewer (IGV 2.16.0) (RRID:SCR_011793) [49]. MACS2 (RRID:SCR_013291) [50] was used to call the significant peaks without building the shifting model with broad peaks (broad-cutoff 0.05) called for H3K27ac, narrow peaks (broad-cutoff 0.05), and input files from respective cells as background. The Bioconductor (RRID:SCR_006442) R package Diffbind 3.10.1 (RRID:SCR_012918) [51] was run on R version 4.3.1 according to the instruction manual to define regions that are differentially enriched by RELA and RELB. ChIP occupancies were evaluated by the computeMatrix tool (RRID:SCR_016366). ChIP-seq profiles and heatmaps were generated from computeMatrix values using the PlotProfiles and PlotHeatmap tools, respectively, on the Galaxy platform (RRID:SCR_006281) [52]. Transcription factor enrichment analyses were performed using ChIP-Atlas (RRID:SCR_015511) [53–55].

### Multiplex immunofluorescence staining

A six-color multiplex immunofluorescence staining was performed using OPAL^TM^ multiplexing method. The staining protocol for FFPE tissue sections was optimized for the simultaneous detection of six antibodies and DAPI for cell nuclear stain. The sections were deparaffinized, rehydrated, subjected to heat-induced epitope retrieval, and incubated with primary and secondary antibodies. The antibodies were visualized using a fluorescent tyramide with Opal 6-Plex Manual Detection Kit (Akoya Biosciences; NEL861001KT). The epitope retrieval and staining process was repeated sequentially for different primary antibodies and fluorescent tyramide combinations. The following primary antibodies with different dilutions were used: CD68 (Cell Marque; 168M-94; RRID:AB_1158188) with 1:500 dilution, Phospho-NF-kB p65 (Ser536) (Cell Signaling; 3033; RRID:AB_331284) with 1:25 dilution, αSMA (Abcam; ab5694; RRID:AB_2223021) with 1:100 dilution, CD31 (PECAM-1) (89C2) (Cell Signaling; 3528; RRID:AB_2223021) with 1:800 dilution, and Pan-Keratin (AE1/AE3) (Cell Signaling; 67306) with 1:50 dilution. Antibodies were visualized with the following tyramide dyes used from the Opal Detection kit (Akoya Biosciences; NEL861001KT): Opal 520, Opal 570, Opal 620, Opal 690, and DIG-Opal 780. Sections were mounted with ProLong^®^ Diamond Antifade Mountant (Thermo Fischer Scientific; P36961). Multiplex-stained slides were imaged using a PhenoImager Fusion system (Akoya Biosciences).

### Statistics and reproducibility

All images presented are representative of at least three biological replicates, except for single-cell migration assays, where graphs represent approximately 3000 cells. Statistical significance was determined using unpaired *t*-tests. Statistical analysis of apoptosis curves was performed by calculating the area under the curve (AUC) followed by unpaired t-tests of the AUC values. Box plots display the 10th to 90th percentile range, with error bars representing the spread of the central 80% of values; these are percentile-based and do not correspond to standard deviation (s.d.), standard error of the mean (s.e.m.), or confidence intervals (c.i.). Upregulated genes and regions were identified using thresholds of log2 fold change >1, false discovery rate (FDR) ≤ 0.05, and BaseMean >10. All graphs and statistical analyses were conducted using GraphPad Prism 10.4.1.

### Schematic images

Model figures were generated using https://www.biorender.com/.

## Supplementary information

Supplemental Materials and Methods

Supplementary Figures S1-5

TNFα-specific and common genes

Western blots used in the study

## Data Availability

All raw and processed data are available under the GEO accession numbers GSE296831 and GSE296832.

## Code availability

Custom software was used to perform automated cell tracking and trajectory analysis and is freely available at: https://github.com/Clark-Lab-Stuttgart/incucyte_cell_tracking.
