# Supplementary material for: Epigenetic context defines the transcriptional activity of canonical and noncanonical NF-κB signaling in pancreatic cancer
Source: Cell Death Discov. 2026 Mar 17;12:152. doi: 10.1038/s41420-026-03019-9 (PMC13039881; doi:10.1038/s41420-026-03019-9)

Supplementary Figure 1

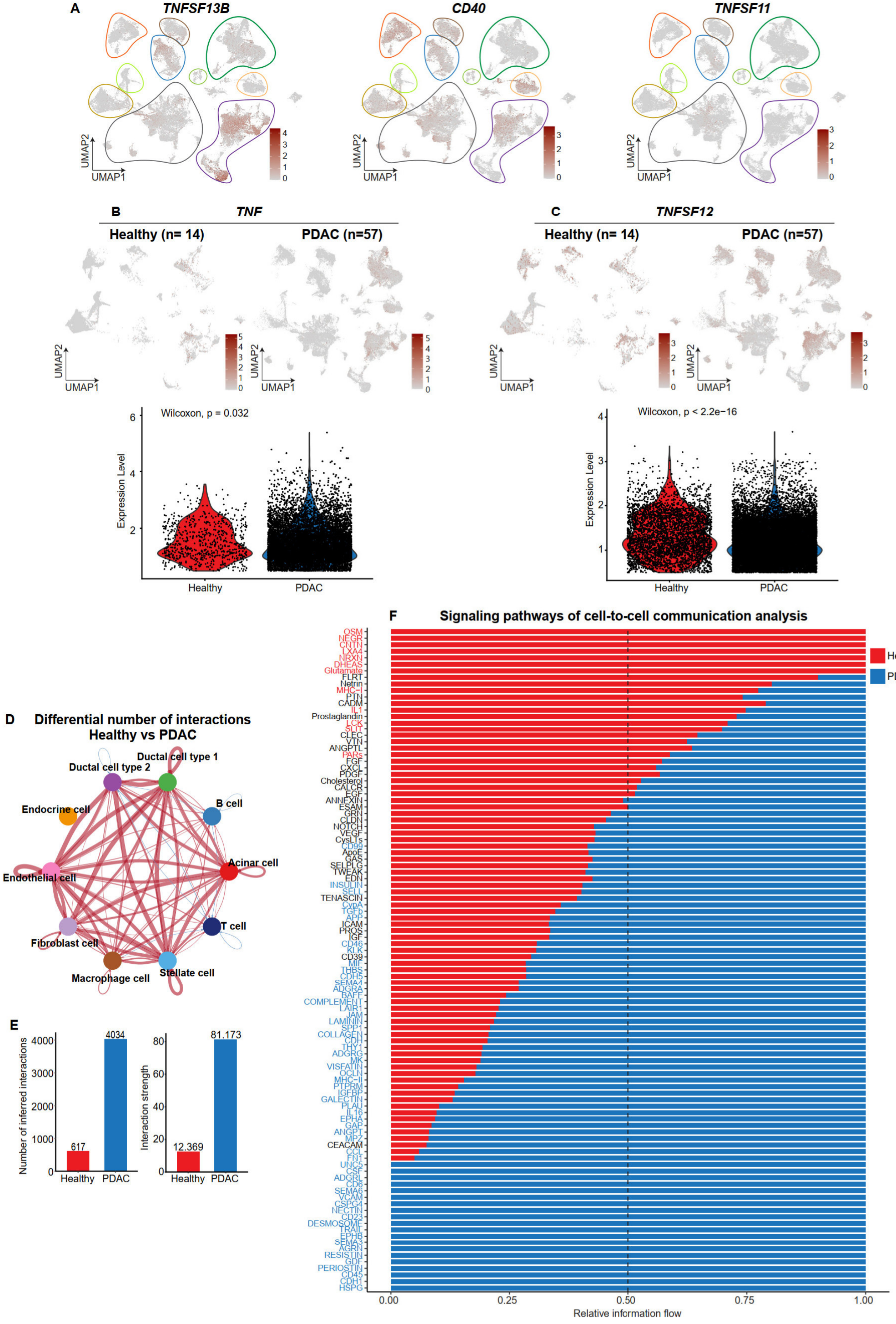

Supplementary Figure 2

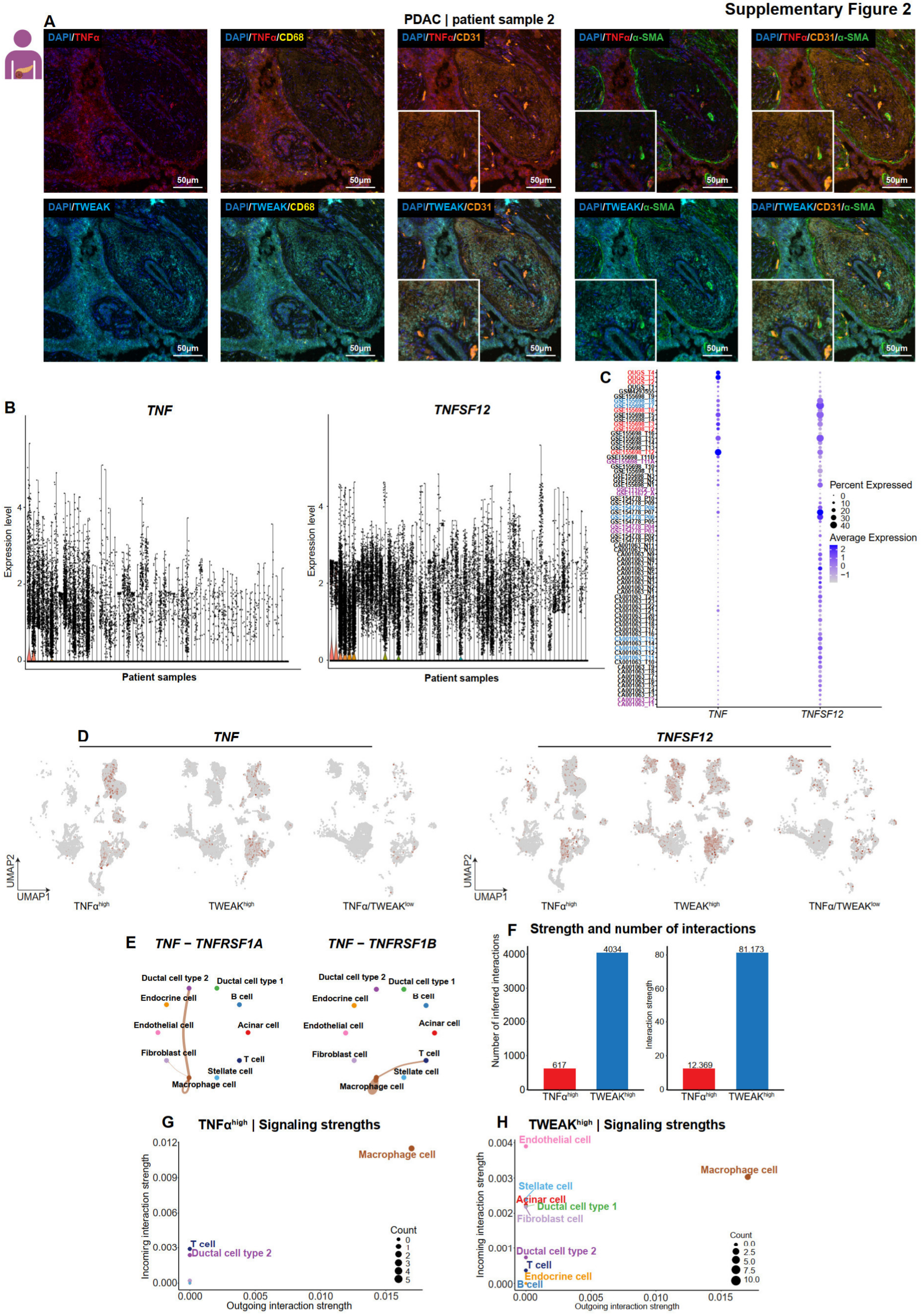

Supplementary Figure 3

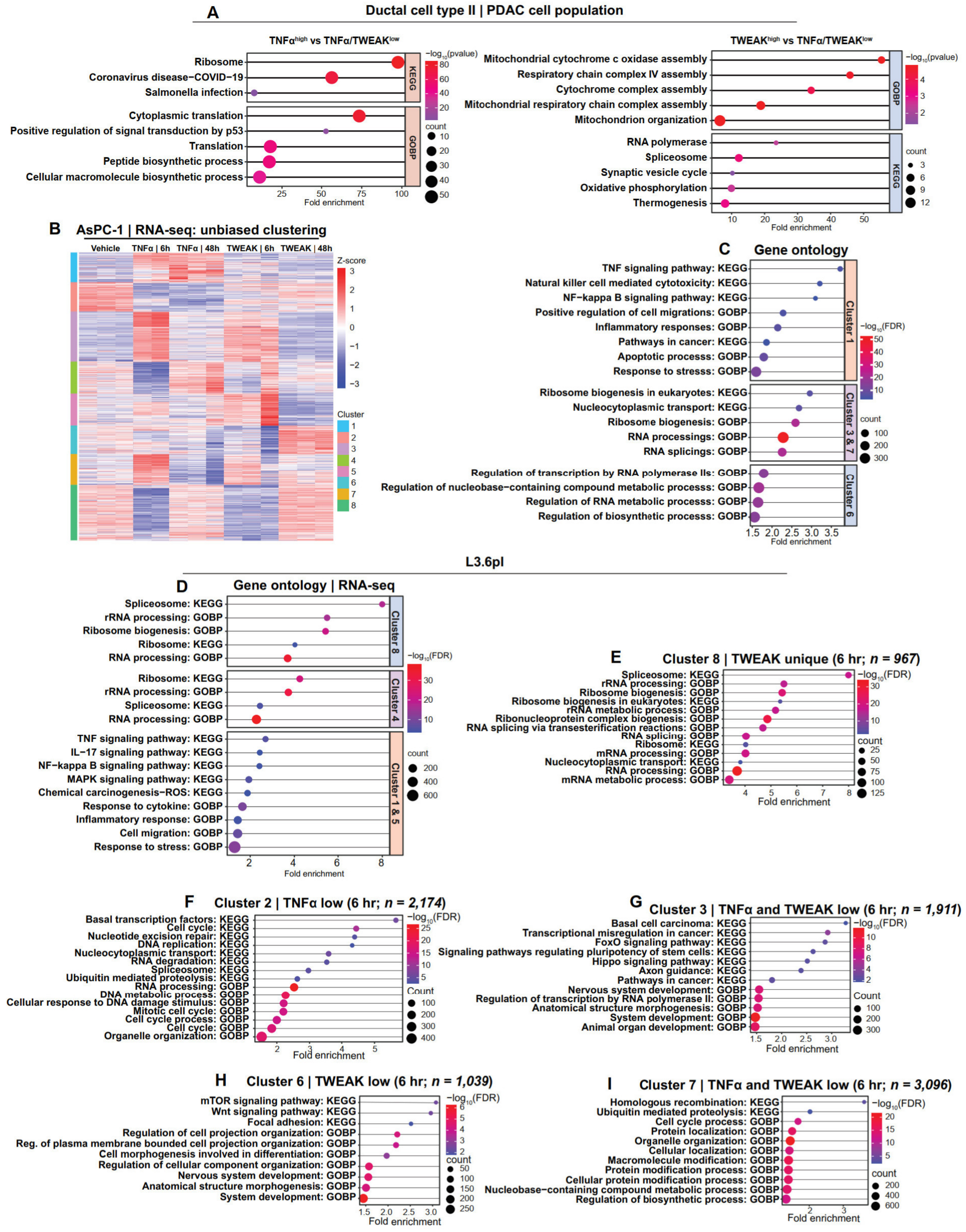

Supplementary Figure 4

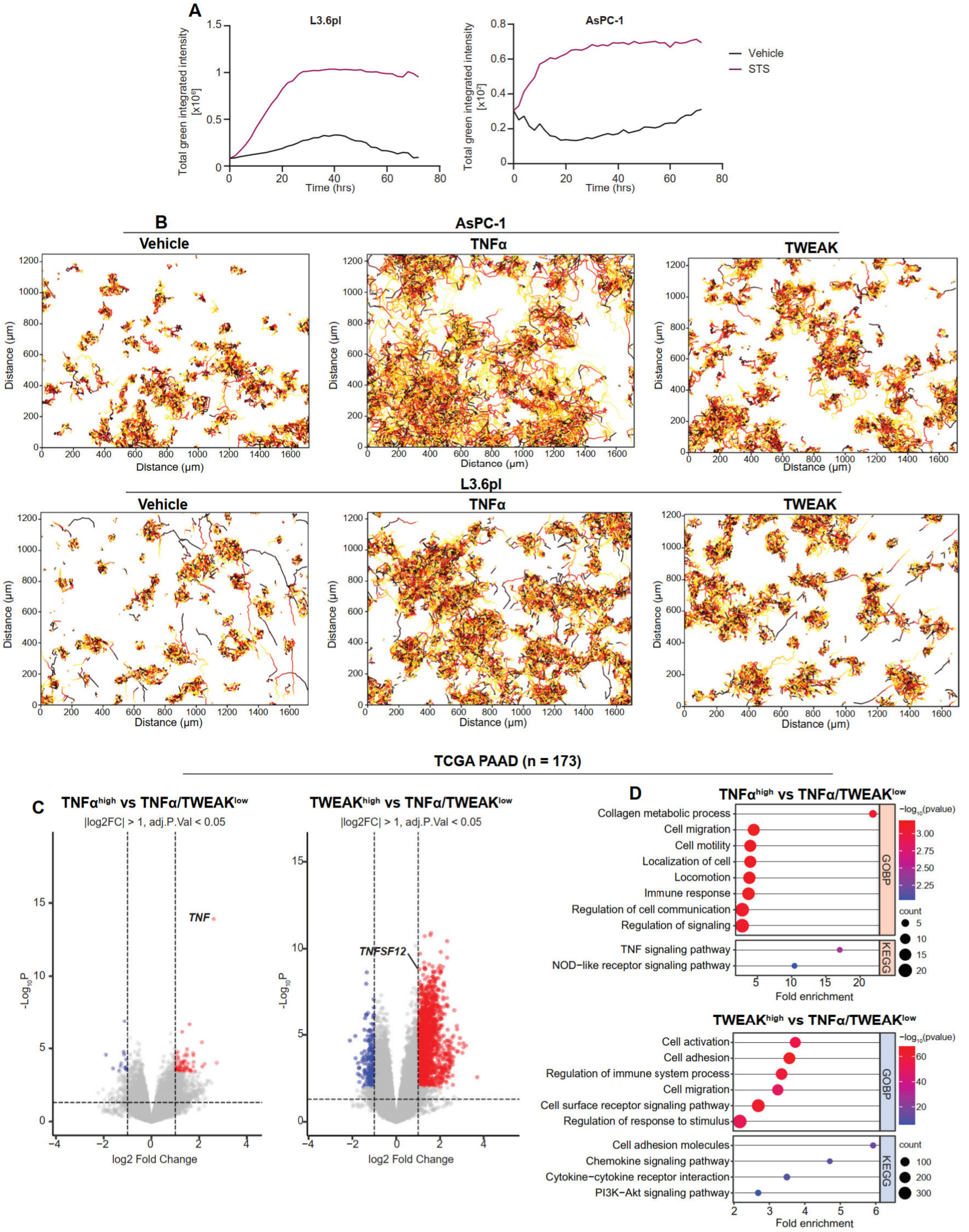

Supplementary Figure 5

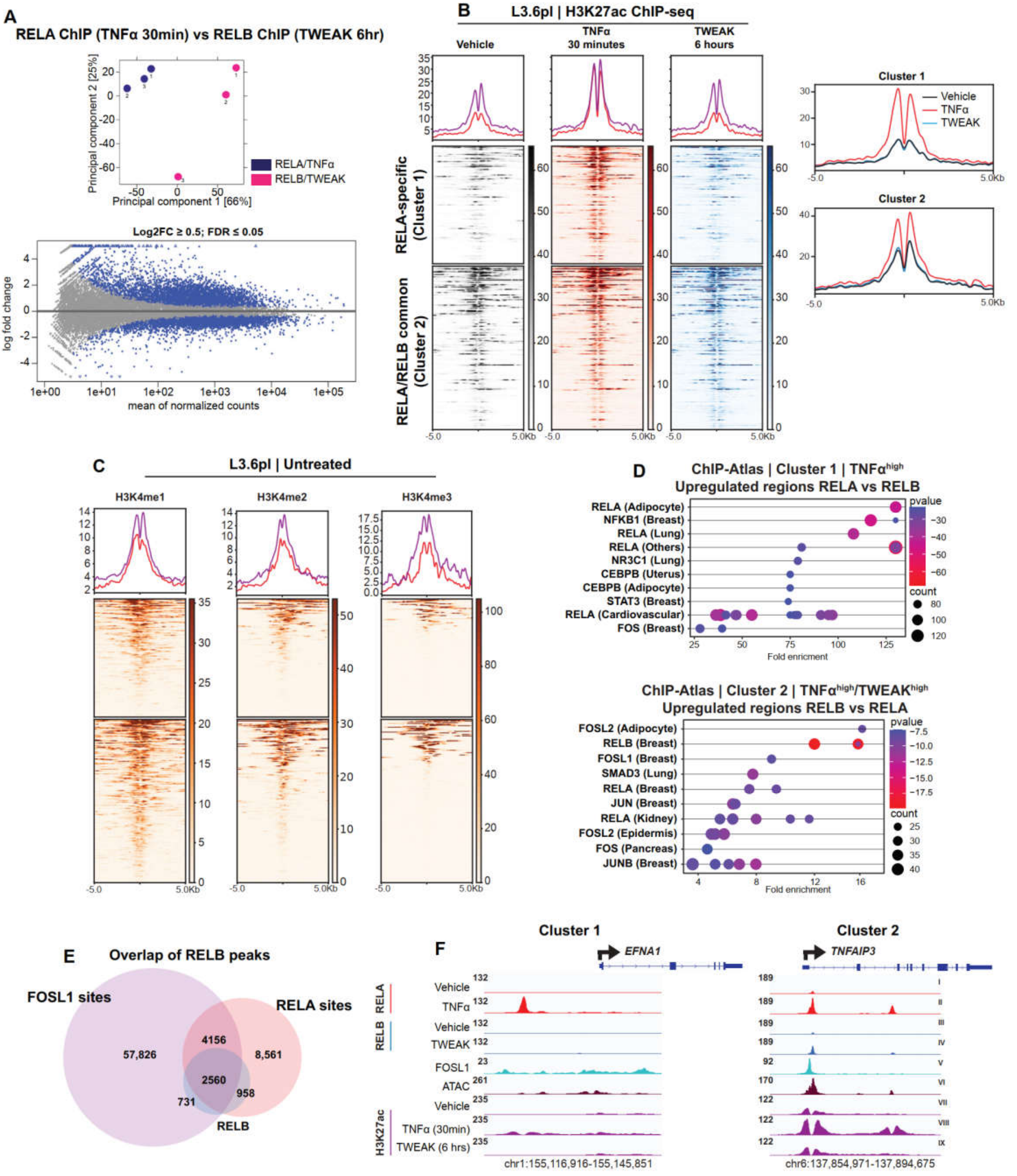

Supplement: Supplementary file 2 — Supplementary Figures S1-5 [file 41420_2026_3019_MOESM2_ESM.pdf]
