# Supplementary figures and images for: Epigenetic context defines the transcriptional activity of canonical and noncanonical NF-κB signaling in pancreatic cancer
Source: Cell Death Discov. 2026 Mar 17;12:152. doi: 10.1038/s41420-026-03019-9 (PMC13039881; doi:10.1038/s41420-026-03019-9)

## TNF $\alpha$

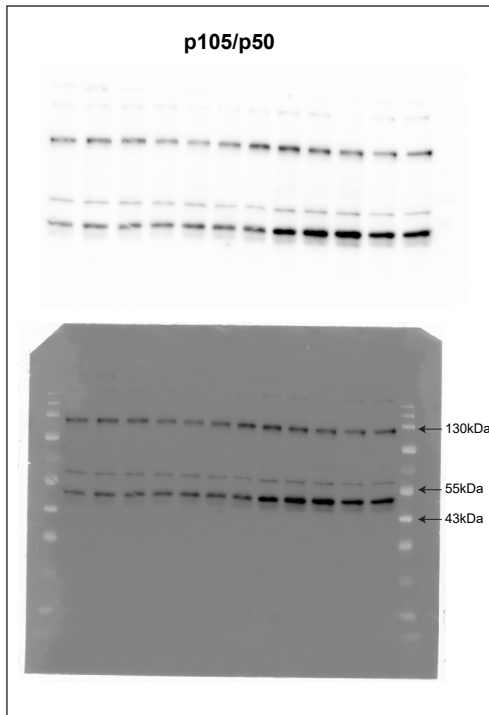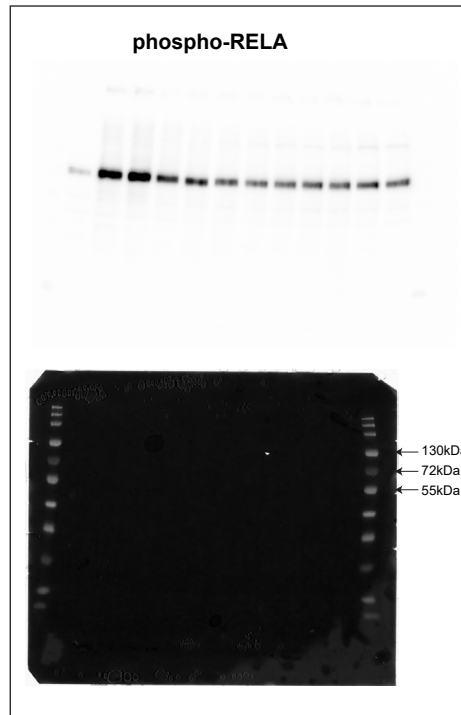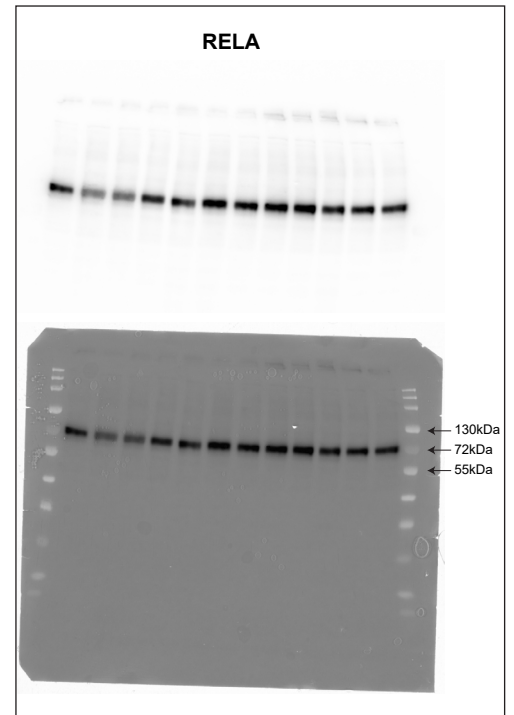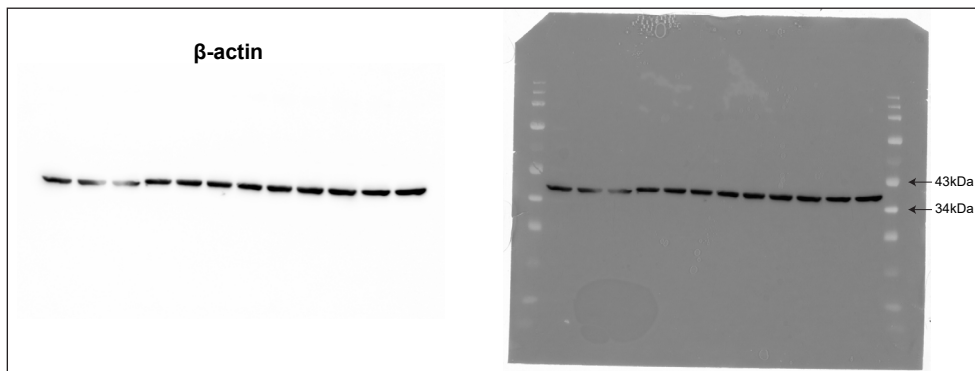

## TWEAK

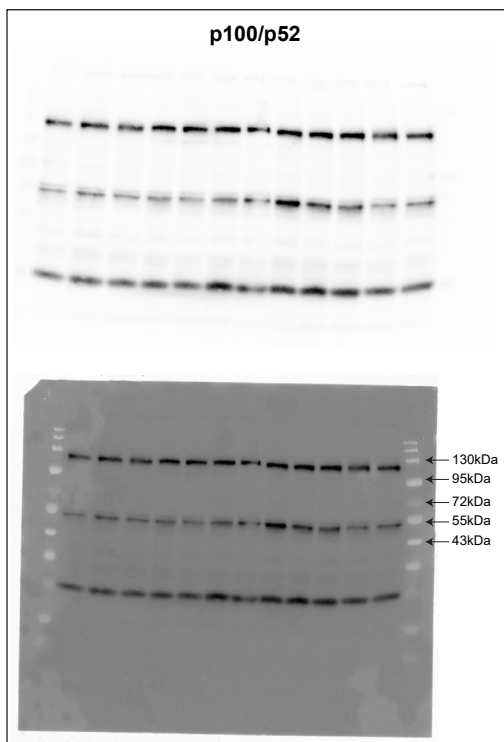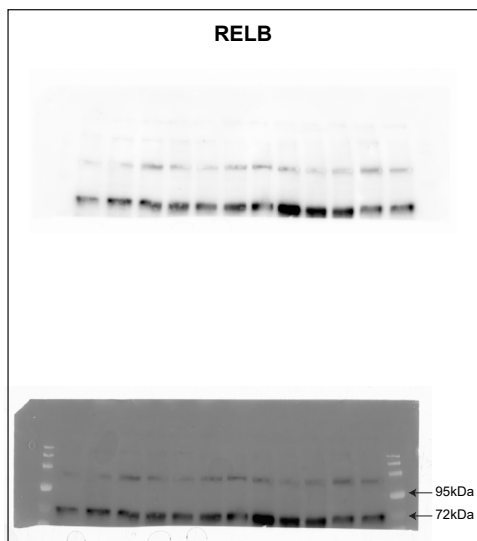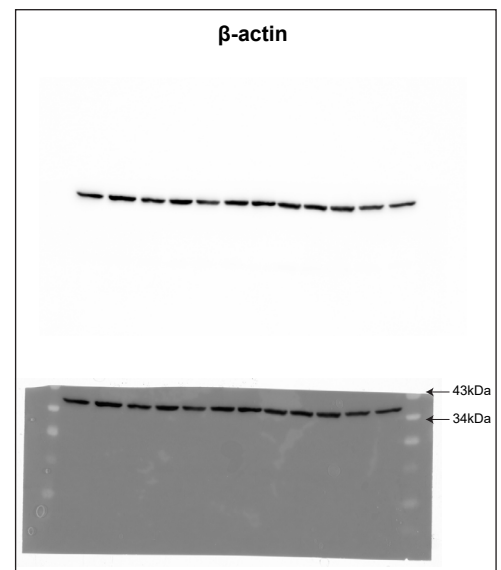

Supplement: Supplementary file 4 — Western blots used in the study [file 41420_2026_3019_MOESM4_ESM.pdf]
